# Stressful task increases drive for thinness and bulimia: a laboratory study

**DOI:** 10.3389/fpsyg.2015.00591

**Published:** 2015-05-06

**Authors:** Sandra Sassaroli, Francesca Fiore, Clarice Mezzaluna, Giovanni Maria Ruggiero

**Affiliations:** ^1^“Studi Cognitivi”, Post-Graduate Cognitive Psychotherapy SchoolMilano, Italy; ^2^“Psicoterapia Cognitiva e Ricerca”, Post-Graduate Cognitive Psychotherapy SchoolMilano, Italy

**Keywords:** bulimia, drive for thinness, experimental task, stress, path analysis

## Abstract

The scientific literature has suggested that stress undergirds the development of eating disorders (ED). Therefore, this study explored whether laboratory induced stress increases self-reported drive for thinness and bulimic symptoms measured via self-report. The relationship between control, perfectionism, stress, and cognition related to ED was examined using correlational methodology. Eighty-six participants completed an experimental task using a personal computer (PC). All individuals completed a battery of tests before and after the stressful task. Analyses showed a significant statistical increase in average scores on the drive for thinness and bulimia measured before and after a stressful task, and path analysis revealed two different cognitive models for the mechanism leading to drive for thinness and bulimia. These findings suggest that stress is an important factor in the development of the drive for thinness and bulimia.

## Introduction

Stressful situations and major life events are widely known to negatively affect eating habits both in humans and in animal models (Wallis and Hetherington, 2004). A reaction to stress occurs when there is a gap between the demands of a given situation and the coping responses of the individual, which in turn reflects the psychosocial resources available to him/her (Lowe and Kral, 2005). The literature suggests that stressful situations may produce a range of effects on eating habits (Steptoe, 1991). Different types of stress factors have been associated with diverse eating responses. For example, mild stress can induce hyperphagia while severe stress can lead to a decrease in food intake (Greeno and Wing, 1994). Individuals facing a stressful event with high anxiety and low social support were more likely to exhibit hyperphagia (Pollard et al., 1995). In addition, a study found that men exposed to simulated stress in a laboratory context ate significantly less than control subjects (Lowe and Kral, 2005). In contrast, another experimental study showed no significant effects of exposure to stress on women's eating behavior, although there was a trend toward a modest increase in consumption of sweet and salty foods (Grunberg and Straub, 1992). Moreover, restrictive eaters who intentionally monitor or restrict food intake to maintain or lose weight tended to consume more energetic and fatty foods in stressful conditions than non-restrained eaters (Heatherton et al., 1991; Polivy and Herman, 1999).

Moreover, the literature has further described multiple, and sometimes contrasting, effects of stress on eating habits as illustrated by the following examples. In an investigation of individuals undergoing acute stress, appetite was found to increase in response to stressful situations, with a higher intake of fatty foods during periods of maximum stress (Lowe and Kral, 2005). On the contrary, other studies have found that severe or chronic stress leads to a reduced intake of food, including fatty foods (Herman et al., 1987; Wallis and Hetherington, 2004). Overall, it seems clear that exposure to stressful situations generally leads to some form of modification of eating behaviors.

In addition, examining the relationship between stress and eating disorders (ED), Ruggiero et al. (2003) reported an association between cognitive variables and symptomatology measures in a real life stressful situation (which was not replicated in non-stressful scenarios). The symptomatology considered included drive for thinness, body dissatisfaction, and bulimia from the ED Inventory (EDI-3), while the cognitive variables included perfectionism (Frost et al., 1990) and self-esteem (Rosenberg, 1965; Vitousek and Hollon, 1990). Ruggiero et al. (2003) found a tendency for drive for thinness and bulimia dimensions to be associated with perfectionism in stressful situations. The body dissatisfaction measure remained unchanged suggesting that in non-clinical females, a stressful situation may increase thoughts of dieting, binge eating and compensatory behaviors. The study supported the hypothesis that stress may be a central factor in transforming a vague insecurity with one's body to a pervasive desire to lose weight and fat. The need for control is another important cognitive aspect of ED. A sense of control is often obtained by continuous monitoring of eating, body weight, and shape (Fairburn et al., 2003). Dietary restrictions enhance the subjective perception of being in control (Slade, 1982). Williams et al. (1990) showed that individuals with any ED perceived a low degree of internal control but high external control exerted by family and society. Hence, in ED low self-esteem combines with a perception of low control over life, which becomes displaced into perception of low control over eating, weight, and fat (Shearin et al., 1994; Fairburn et al., 1998; Masheb and Grilo, 2002; Eiber et al., 2005). Serpell et al. (1999) and Waller (1998) have shown that gaining a sense of control and pride in controlling food intake combats the feeling of being taken over by thoughts of food or of lacking control over personal thoughts, eating, and weight.

Based on previous findings, we designed a study to induce a stressful situation in a laboratory setting to determine, in a highly controlled environment, if and how stress affects beliefs related to nutrition as measured by two subscales (drive for thinness and bulimia) in a sample of post-graduate psychology students. Stress induction in a laboratory setting distinguishes this design from previous studies. Another aim was to investigate whether stress can predispose a particular food trend (and related cognitive beliefs) in relationship to control and perfectionism using Structural Equation Modeling (SEM) methodology.

## Methods

### Instruments

Before participating in the experiment all subjects completed SCID I and II to assess inclusion criteria. The General Health Questionnaire (GHQ; Goldberg, 1972) is a widely used screening tool. It is a 12-item self-administered measure of psychological well-being aimed to detect stress symptoms that may signal distress. This measure was important because it allowed us to exclude people who were suffering from psychological distress from the study. The GHQ has demonstrated good psychometric validity (α = 0.95) and reliability (α = 0.83).

Self-report subscales from EDI-3, drive for thinness and bulimia, were used to assess cognitive and behavioral dimensions of ED (Garner, 2004). Drive for thinness is useful in screening for ED. It measures a core feature comprising excessive attention to diet and fear of weight gain (Garner, 1991, 2004; Engström et al., 1999; Abood and Black, 2000). This subscale is based on the clinical conceptualization by Bruch (1973) and Russell (1970). The bulimia subscale assesses the tendency to worry about and engage in uncontrollable binge eating, which is one of the defining features of bulimia and the binging-purging subtype of anorexia (Bruch, 1973; Garner, 2004).

The use of individual subscales from a questionnaire is accepted practice, if reliability indexes are satisfactory. For example, Yoon et al. (2010) used only one subscale of the Maslach Burnout Inventory and Archer and Thanzami (2009) also used only one subscale of the Narcissistic Personality Inventory. In addition, Ruggiero et al. (2003), and Sassaroli and Ruggiero (2005) used the same three subscales mentioned above from the EDI-3. In the current study, the Cronbach's alpha values for each subscale of the EDI were acceptable, all above 0.7. The Anxiety Control Questionnaire (ACQ; Rapee et al., 1996) assesses perception of control over emotional reactions and external threats. It is designed to detect pathological perceptions of low control as well as an exaggerated fear of losing control. The ACQ is a 30-item questionnaire providing a total score which is the sum of the two subscales: the 16-item event subscale and the 14-item reactions subscale. Participants responded on a 6-point Likert scale. Lower scores correspond to individuals with an emotional disorder. We considered the ACQ total score because the psychometric properties of the composite score are stronger than those of the subscales taken individually (Antony et al., 2001). The total score has been shown to be internally consistent, with high test-retest reliability, and is a valid measure for discriminating between anxious and non-anxious individuals (Russell, 1970).

Therefore, examination of the individual ACQ subscales would have unnecessarily complicated the statistical analyses and weakened the psychometric qualities of the instrument.

The Multidimensional Perfectionism Scale (MPS; Frost et al., 1990) is a 35-item self-report questionnaire which measures six dimensions of perfectionism, including concern over mistakes, that are based on Frost's theoretical model of perfectionism (Parker and Adkins, 1995). In this study, we used the concern over mistakes subscale (CM) of the MPS instead of the perfectionism subscale of the EDI-3 because it is specifically designed to detect maladaptive levels of perfectionism when present. Psychometric studies have shown that the MPS and the CM subscales have adequate reliability (Cronbach's α was higher than 0.7) (Frost et al., 1990; Parker and Adkins, 1995). Internal consistency measured by Cronbach's α for each administered test was between 0.71 and 0.85 in this sample.

### Participants

A total of 128 healthy participants were recruited at the post-graduate school Studi Cognitivi in Milan and there was no financial incentive to participate. All participants were university graduates of Italian nationality (77 from Northern and 51 from Southern Italy); 47 were married, 57 were in a committed relationship/dating and 24 were single. Eleven participants were excluded from the trials because they obtained high scores on the GHQ in the first administration indicating they were already stressed (Cut off = 11; Goldberg, 1972); and 31 individuals were excluded due to the presence of an ED. Twenty-four participants had sought psychological help for an anxiety disorder in the past, 19 were currently in psychological therapy for an anxiety disorder, and 43 had never received psychological therapy. The BMI mean of participants was 22.56 (*SD* = 2.01).

Additional criteria for inclusion in the study were minimum age of 18 years, adequate written language abilities, and no co-morbid personality disorder. Thus, we obtained a total sample of 86 subjects aged between 21 and 39 years, matched for gender [41 males (mean age = 28.50; *SD* = 4.67) and 47 females (mean age = 39.15; *SD* = 4.58)], in order to control for gender effects. Individuals that obtained a GHQ score between 2 and 6 were considered non-stressed and, for this reason, suitable participants for the experiment. The study was described to participants as an investigation of the role of stress on beliefs about eating. Following a brief introduction of the project and filling out the informed consent, participants were instructed, both verbally and in written form, to complete the self-report instruments and cognitive task. The study was approved by the Ethical Committee of the “Studi Cognitivi” cognitive psychotherapy school in Milan.

### Experimental procedures

After completing the questionnaires, participants were asked to perform a cognitive visuo-spatial working memory task on a personal computer (PC). We tested all individuals after breakfast to avoid effects of appetite on cognitive task.

The visuo-spatial task was a modified version of the n-back paradigm aimed at inducing cognitive interference (Owen et al., 2005). The literature suggests that cognitive interference induced by this task is a very demanding experience for the human mind and has frequently been used in order to artificially induce stress in the laboratory (Owen, 2004). The reason is that normal subjects are highly confused by a task which uses geometrical figures resulting from an n-back paradigm (Owen, 2004).

**Figure 1:**
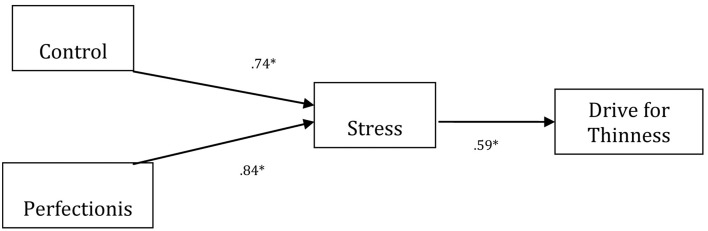
**The estimated model of Perfectionism, Control, and Stress on Drive for Thinness.** Please note that standardized coefficients are presented. For ease of presentation, error terms were omitted. ^*^p < 0.05. Fitting index data: (χ^2^ = 16,769.25; gdl = 387; *p* < 0.001; CFI = 0.960; NNFI = 0.956; GFI = 0.967; AGFI = 0.968; RMSEA = 0.034).

During the task, each participant was shown a series of images (squares) displayed serially at the center of a computer screen. The task was divided into three experimental conditions of increasing difficulty called 0-back, 1-back, and 2-back, respectively. The 0-back condition, which served as a baseline, consisted of deciding whether each stimuli corresponded to a specific target. The 1-back condition consisted of deciding whether each stimuli corresponded to the penultimate one in the sequence. The 2-back condition consisted of deciding whether each stimuli corresponded to the antepenultimate one in the sequence. In each condition, six blocks of 36 trials were conducted for a total of 216 trials. The trial sequence comprised a 500 ms stimuli display (total of 696 stimuli) followed by a 2500 ms fixation cross point (Figure 1). One third of the items were selected as targets, randomly arranged in each block. Within each condition, block and trial order were consistently maintained for all participants. Each participant completed the task 6 times.

The task was computerized and built using Matlab Software. The software programme was run on a PC that recorded each trial, time, and accuracy of responses. Participants could give their answer through mouse's key. In particular, left button corresponded to a positive response and the right button to a negative response. In order to investigate the effects of experimentally-induced stress on cognition regarding eating, weight, and body shape, participants completed the drive for thinness and bulimia subscales of the EDI again at the end of this task.

### Instrumental measures of stress

Prior to conducting the experimental task we verified if the cognitive task actually stressed the individuals. Thus, we divided the 20 subjects into two groups: the first one performed a visuo-spatial working memory task and the second one performed a control task which required subjects to press the “enter” key on the PC every time they saw a geometric figure. An increase in GHQ score was found in the first group that performed the cognitive task [*t*_(1)_ = 2.17; *p* < 0.05], but the second group who performed a control task, did not have a significant increase in GHQ score [*t*_(1)_ = 3.45; *p* < 0.05]. Then, we began the experiment.

The reaction time and accuracy of subjects during the working memory task were used to measure stress. These were measurements of subjects' cognitive ability in overcoming cognitive interference. In total, participants had a high score in reaction time but low accuracy. In order to ensure that participants were stressed we administered the GHQ before and after the cognitive task. A *t*-test analysis confirmed that participants exposed to the experimental task were stressed [*t*_(1)_ = 2.67; *p* < 0.05].

### Data analytic strategy

Examinations of skewness and kurtosis, as well as tests of normality, revealed that distributions of experimental variables were normal. As a consequence, a series of multivariate analysis were conducted. To identify significant differences in drive for thinness and bulimia, time T0 pre-cognitive task administration of the two subscales of EDI were compared to time T1 post-cognitive task administration of the same subscales using a MANOVA to test whether the experimental task significantly increased scores of self-reported measures of ED. The same hypothesis was tested in males and females separately, (conditions: Males and Females) × 2 (conditions: Time T0 and T1) × 2 (conditions: Drive for Thinness and Bulimia).

In order to test whether stress mediated the relationship between the cognitive correlates of ED (i.e., perfectionism, control, and bulimia or anorexia) we used SEM. For model estimation, the LISREL SOFTWARE procedure was used; the method of estimation was Maximum Likelihood (Jöreskog and Sorbom, 1989). Additional fit indices were also examined including: Comparative Fit Index (CFI; Bentler, 1989), the Non-Normed Fit Index (NNFI; Tucker and Lewis, 1973; Bentler and Bonnett, 1990), the Goodness of Fit Index (GFI; Jöreskog and Sorbom, 1989), the Adjusted Goodness of Fit Index (AGFI; Jöreskog and Sorbom, 1989) and the Root Mean Squared Error of Approximation (RMSEA; Steiger and Lind, 1980; Steiger, 1990). The RMSEA (acceptable fit: 0.05–0.08; good fit: 0–0.05) the CFI and NNFI (acceptable fit: 0.95–0.97; good fit: 0.97–1) and the GFI and AGFI assess the amount of variance and covariance explained by the matrix that is reproduced, for which values greater than 0.90 indicate a reasonable fit to the data (Hu and Bentler, 1999).

## Results

A significant difference was found in both drive for thinness and bulimia subscales of EDI at T0 pre-cognitive task administration vs. T1 post-cognitive task administration (Table 1). In addition, the differences were also significant when we analyzed males and females separately (Table 1). Even after controlling for psychiatric diagnosis [*F*_drive for thinness(1,84)_ = 18.56; *p* < 0.05; *F*_bulimia(1,84)_ = 19.46; *p* < 0.05] and socio-demographic variables [*F*_drive for thinness(1,84)_ = 18.45; *p* < 0.05; *F*_bulimia(1,84)_ = 21.33; *p* < 0.05] the results stayed the same.

**Table 1:** **Descriptive statistics and between group comparisons on outcome measures at pre- and post-administration tests**.

**Measure**	**Pre-administration of cognitive task**	**Post-administration of cognitive task**	**Multivariate analysis**		
**All subjects**	**Time T0 *M*(*SD*)**	**Time T1 *M*(*SD*)**	**Main effect of time *F*_(1, 84)_**	**Main effect of condition *F*_(1, 84)_**	**Time × condition interaction *F*_(1, 84)_**
Drive for Thinness	11.6 (7.07)	16.02 (7.64)	9.87^**^	29.96^***^	36.78^***^
Bulimia	5.25 (1.79)	8.65 (4.76)			
**Females**	**Time T0 *M*(*SD*)**	**Time T1 *M*(*SD*)**	**Main effect of time *F*_(1, 39)_**	**Main effect of condition *F*_(1, 39)_**	**Time × condition interaction *F*_(1, 39)_**
Drive for Thinness	14.7 (6.76)	22.15(3.76)	17.442^*^	11.93^***^	0.42
Bulimia	5.15 (1.81)	9.35 (5.59)			
**Males**	**Time T0 *M*(*SD*)**	**Time T1 *M*(*SD*)**	**Main effect of time *F*_(1, 39)_**	**Main effect of condition *F*_(1, 39)_**	**Time × condition interaction *F*_(1, 39)_**
Drive for Thinness	8.50 (6.04)	9.90 (5.30)	19.37^*^	4.61^**^	8.11^**^
Bulimia	5.85 (1.81)	7.95 (3.77)			

M = mean, SD = standard deviation, F = Fisher index of MANOVA statistics,

* p < 0.05;

** p < 0.01;

*** p < 0.001.

Additionally, we tested for possible mediation effects of cognitive variables (i.e., control and perfectionism) between stress and EDI variables (i.e., drive for thinness and bulimia). SEM results demonstrated that the variables control, perfectionism, and stress predicted drive for thinness (Figure 1). The paths from perfectionism to stress (β = 0.125; *p* < 0.05) and from control to stress were significant (β = 0.134; *p* < 0.05), and there was a positive association between stress and drive for thinness (β = 0.156; *p* < 0.01).

**Figure 2:**
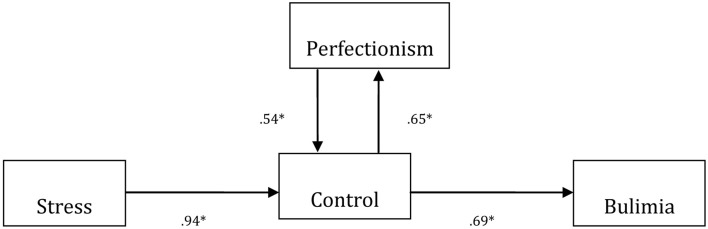
**The estimated model of Perfectionism, Control, and Stress on Bulimia**. Please note that standardized coefficients are presented. For ease of presentation, error terms were omitted. ^*^p < 0.05. Fitting index data: (χ^2^ = 16,769.25; *df* = 387; *p* < 0.01; CFI = 0.957; NNFI = 0.956; GFI = 0.975; AGFI = 0.978; RMSEA = 0.027).

We also tested the hypothesis that control, perfectionism, and stress predicted bulimia. The results are shown in Figure 2. We also obtained fit indexes for the data. The path from stress to control was significant (β = 129; *p* < 0.05) and there was an interaction between control and perfectionism (β = 0.112; *p* < 0.05) as well as a positive association between control and bulimia (β = 231; *p* < 0.01).

In conclusion, stress was an endogenous variable in the model related to drive for thinness and an exogenous factor in the model related to bulimia. Possible clinical interpretations of these models are discussed in the following section. Alternative models in which stress played a different role (either exogenous in the model related to drive for thinness or endogenous in the model related to bulimia) did not provide adequate fit.

## Discussion

The results of the present study can be interpreted as providing possible support to the hypothesis that stress is a factor related to some cognitive features widely associated with ED. In fact in our sample we found the same effect of stress on the variables being studied even after controlling for psychopathological illness. These variables included a desire to become thinner, fear of being too fat, dieting ideation and bulimic impulses of binging and purging. This study suggests that stressful situations may be a prodromal factor which plays a role in the mechanism leading to the development of an ED. These results were also in line with previous studies (Ruggiero et al., 2003; Sassaroli and Ruggiero, 2005) which have already shown the role played by stress in ED. Moreover, experimental demonstration of this mechanism more reliably supports the importance of stress in the origin of ED tendencies.

The current study has partially confirmed the importance of the impact of stressful situations on the association between psychological dimensions and measures of cognitive beliefs in ED. Moreover, this study demonstrated that the influence of stress was present not only in females but also in males whereas previous studies only found effects of stress in females. This suggests that laboratory-induced stress is more controlled for the study of stress than quasi-experimental designs using real-life stressful events. Given the results indicated in Figure 1, the cognitive task used to engender stress in individuals offers a plausible psychological mechanism. This stress was shown to increase drive for thinness and bulimic impulses while the GHQ score confirmed the realness of the stressful condition.

Path analysis revealed two different cognitive models for the mechanism involved in drive for thinness and bulimia. In the first case, control and perfectionism affected sensitivity to stress, which in turn affected drive for thinness (Figure 1). In the second case, the path starting from stress affected control, which in turn affected bulimia. Furthermore, control was related to perfectionism via a bidirectional effect (Figure 2). In the end, these results suggest that stress can directly affect the cognitive beliefs related to drive for thinness and bulimia and is therefore a proximal factor in the case of drive for thinness, with an indirect effect in the case of bulimia. This confirms a similar finding in a recent study by Sassaroli and Ruggiero (2005). These different models of operation, in our opinion, derive from two different cognitive structures present in individual's cognitive beliefs concerning ED. In fact, for example, in clients with anorexia, stress exacerbates the cognitive variables of perfectionism and control, which were already present in the patient's personality structure, while in bulimia, stress leads to perfection and control mechanisms that were not previously manifest. To confirm this finding, when other models were tested with our data, the results were not significant.

Our results have possible clinical implications when treating drive for thinness: interventions should be aimed at recognizing the use of perfectionism and control when experiencing negative events which produce restrictive behaviors. Meanwhile, when treating bulimic behaviors, the therapist may presume that control is the initial psychological attitude used by patients to cope with a stressful situation, which could be influenced by perfectionism personality traits. In this case, the therapist should encourage the patient to recognize his/her vulnerability as a consequence of the tendency for perfectionism.

The limitations of this study include the small sample size and the use of partial clinical participants. Therefore, in order to generalize the results, future research should increase the size of the sample and perform the same study in a group of clinical individuals only with different psychiatric diagnoses.

### Conflict of interest statement

The authors declare that the research was conducted in the absence of any commercial or financial relationships that could be construed as a potential conflict of interest.
